# Psychosocial problems of Pakistani parents of Thalassemic children: a cross sectional study done in Bahawalpur, Pakistan

**DOI:** 10.1186/1751-0759-6-15

**Published:** 2012-08-01

**Authors:** Kashif Aziz, Breera Sadaf, Sadia Kanwal

**Affiliations:** 1 Department of Community Medicine, Quaid e azam Medical College, c/o Dr Shafqat Nazeer 27-Riaz Colony, Bahawalpur, Pakistan; 2 Department of Community Medicine, Quaid e azam Medical College, Bahawalpur, Pakistan; 3 Department of Psychology, Islamia University, Bahawalpur, Pakistan

**Keywords:** Parents, Psychological morbidity, Thalassemia, Social relationship, Adjustment disorder, Pakistan

## Abstract

**Background:**

Thalassemia is a blood disorder passed down through families (inherited) in which the body makes an abnormal form of hemoglobin. This disorder results in excessive destruction of red blood cells, and there is no effective treatment. Patients require lifelong blood transfusion, usually started within 6 to 12 months of birth of patient, which on other hand has its own complications. It is a chronic disease that manifests so early in life that it leads to psychological and social problems for parents . We focused on parents to assess the impact of their child’s disease.

**Objective:**

To determine the psychosocial problems of parents of thalassemic children.

**Methods:**

This cross sectional study was conducted among the parents of thalassemic children attending THALASSEMIA CENTRE, BAHAVAL VICTORIA HOSPITAL (BVH), BAHAWALPUR, PAKISTAN during the year 2011. A self designed questionnaire was used that contained questions regarding psychological and social aspects. Patient Health Questionnaire-9 (PHQ-9) was used to assess the depression of parents of thalassemic children.

**Results:**

Of the 100 parents interviewed, the majority were mothers (71%) , with a mean age of 32 ± 8.07 years for both sexes. 29 percent of the parents had moderate to severe depression, 16 percent had sleep disturbances. 56 percent were downgraded by relatives. There was a significant relationship between respondent education and depression (p < 0.05).

**Conclusion:**

A substantial number of parents have psychosocial problems due to the disease of their child. Parent counseling is needed on regular basis.

## Background

Worldwide, thalassemia poses a serious public health problem due to the high prevalence. It extends from the Mediterranean basin and parts of Africa, throughout the Middle East, the Indian Sub-continent, South-East Asia, Melanesia and into the Pacific Islands, with reported rates ranging from 2% to 25% [1]. Each year, 50,000 to 100,000 children die of thalassemia major in low and middle income countries, while about 7% of the world's population is a carrier of a hemoglobin disorder [1].

In Pakistan, thalassemia is seen in almost all parts of the country [2]. Every year, 5000 babies are born with thalassemia - five out of 100 persons are currently suffering, and around 8 million of the population are carriers [3]. The average life expectancy for thalassemia is about 10 years [2].

The more severe forms are beta-thalassemia major, which warrants regular blood transfusion at an early age, and thalassemia intermedia which presents later and requires less frequent transfusions. The aim of regular blood transfusions is to eliminate the primary complications of severe thalassemia by ameliorating anemia and suppressing erythropoiesis. Patients are usually transfused at an early age. The chronicity and complications of thalassemia affect the quality of life of victims and parents and cause physical, psychological, and economic problems [4].

As there is no definitive cure for this disease, the majority exclusively depend on blood transfusions as a treatment option that creates a burden not only on health system but also on the affected families, who are vulnerable to, social, and psychological problems [5,6]. Various Quality of Life (QOL) studies conducted worldwide on thalassemia indicated poor indicators for the sufferers [7]. However, literature review has revealed a lack of studies that address the impact of a diseased child on the life of the parents of thalassemic children in Pakistan. The present study aimed to clarify the psychosocial problems experienced by parents of Pakistani thalassemic children.

## Methods

This Cross sectional Study was conducted in the Thalassemia Centre of Bahawal Victoria Hospital in Bahawalpur, Pakistan. Ethical Approval of this study was given by the hospital ethics committee. The study was carried out from March 2011 to August 2011. The non -probability convenient sampling technique was used. All Parents who brought their children for blood transfusion were approached during the study period from 9 am to 1 pm 5 days a week, excluding Sunday and public holidays. Among them, only those parents who fulfilled the inclusion criteria were included in the study. The criteria were:

 (a) established diagnosis of thalassemia of their child and the child was registered with the Thalassemia centre for regular blood transfusion.

 (b) parents who provided consent for the study.

Only the mother or father of the child was included in the study. Grandparents and step mothers or step fathers were excluded from the study, and mothers with stepfathers were also excluded as they could have confounded the result.100 parents fulfilled the above criteria. Informed consent was taken from the parents who participated in the study. Data was collected on a questionnaire comprising 13 questions that was self designed to assess the psychological and social problems. In it following parameters were studied.

 1. How much has this disease affected their normal life routine, like eating and sleeping habits?

 2. Effect of disease on economic status.

 3. Effect of disease on social life, i.e. relation with spouse and relatives.

 4. Effect of disease on family size.

 5. Desire to terminate the pregnancy if it would have been diagnosed prenataly.

 6. Use of medicine to relieve tension due to this disease.

 7. Would allow their children to marry a cousin?

The questionnare is attached as Additional file 1.

This questionnaire was translated into the local language.

PHQ-9 was used to assess the degree of depression among these parents, and depression was scored according to the scoring card of the questionnaire. PHQ 9 was also translated into the local language. First, a pilot survey was done using these translated questionnaires. After testing these two questionnaires in a pilot survey, they were introduced for use in this study. The PHQ-9 questionnaire has 9 questions. It is directly based on the diagnostic criteria for major depressive disorder in the Diagnostic and Statistical Manual Fourth edition (DSM-IV). These questions are asked to assess symptoms and functional impairment due to depression within the last one month. For each question, four categories are given that either the situation had affected ‘not at all’, affected ‘several days in month’, ‘more than half the days in a month’, or ‘affected every day’. For each question, if ‘not at all’ was selected a 0 score was given, if ‘several days’ was selected a 1 score was given, if ‘more than half days’ was selected a 2 score was given, and if ‘every day’ was selected a 3 score was given. The scores of all 9 questions were added to give the total PHQ-9 score. On the basis of the scoring card, 5 categories were formed as follows.

Total score Depression severity.

1 to 4 minimal Depression.

5 to 9 Mild Depression.

10 to 14 Moderate Depression.

15 to 19 Moderately severe depression.

20 to 27 Severe Depression [8].

Data was entered, stored and analyzed with the help of SPSS software, version 17.0. Frequencies were tabulated for demographic data as well as for psychosocial issues. Cross tabulation using Chi Square was done for educational level, inability to attend social gatherings, and drug intake of parents for their depression. p < 0.05 was considered statistically significant.

## Results

Of the 100 parents, 71 were mothers, with a mean age of 32 ± 8.07 years for both sexes. Of them 43% were illiterate. 34 percent of the affected children were female. The demographic details are given in Table 1.

**Table 1:** Demographic data of the parents of thalassemic children

**Demographic Data**				
**1**	**Respondent relation to affected child**		**Frequency**	**Percent**
		Mother	71	71
		Father	29	29
**2**	**Respondent Age**			
		15 yr to 20 yr	2	2
		21 yr to 25 yr	7	7
		26 yr to 30 yr	34	34
		31 yr to 35 yr	17	17
		36 yr to 40 yr	26	26
		Above 40 yr	14	14
		Mean age	32 ± 8.07 yr	
**3**	**Father’s Educational status**			
		Illiterate	43	43
		Undermatric	34	34
		Matric	13	13
		Undergraduate	2	2
		Graduate	3	3
		Postgraduate	2	2
**4**	**Mother’s Educational Status**			
		Illiterate	75	75
		Undermatric	14	14
		Matric	9	9
		Undergraduate	1	1
		Graduate	0	0
		Postgraduate	0	0
**5**	**Family Income Status in PKRs**			
		Less than 5000	37	37
		5000 to 10000	40	40
		10000 to 20000	13	13
		More than 20000	10	10
**6**	**Gender of Affected Children**			
		Male	66	66
		Female	34	34
**7**	**Birth Order of Affected children**			
		1^st^	38	38
		2^nd^	25	25
		3^rd^	9	9
		4^th^	14	14
		5^th^	10	10
		6^th^	3	3
		7^th^	0	0
		8^th^	1	1

Table 2 lists the responses given by parents to the questions. These included questions relating to psychological and social aspects of their life. 56 percent of parents reported that this disease excessively affected their economic status. 27 percent were completely unable to go to social gatherings. Only 8 percent reported that the disease of their child had not affected their daily routine work. 7 percent of the parents reported that is the disease was not related to their marriage to a relative, but still they had a thalassemic child. 23 percent had conflicts with their spouse due to their child’s disease. The majority of the parents (76%) said that they would have terminated their pregnancy if thalassemia had been diagnosed prenatally. 29 percent of the parents had moderate to severe depression. Only 3 percent had severe depression. More severe depression was found in the mothers, as compared to fathers. No father was found to have moderate to severe depression, while 12 percent of mothers had moderate to severe depression. The mean depression score of mothers as assessed by PHQ-9 was 8 ± 5.5, while for fathers this value was 5 ± 4.9.

**Table 2:** Response frequencies of parents (as a percentage) and severity of depression

**Variable**	**Response of parents**			
	**Not at all**	**Slightly**	**Moderately**	**Excessively**
**Unable to concentrate on daily work**	8	35	36	21
**Affected eating habits**	34	41	13	12
**Affected sleep**	34	27	23	16
**Affected economic status**	9	8	27	56
**Unable to attend social gatherings**	22	28	23	27
**Variable**	**Response of parents**			
	**yes**		**No**	
**Affected relation with spouse**	23		77	
**Downgraded by relatives**	56		44	
**Take drug to relieve tension**	23		77	
**Would have terminated this pregnancy if it had been diagnosed prenatally**	72		28	
**Affected respondent’s desired family size.**	44		56	
**Is this your family marriage**	93		07	
**Would do cousin marriage of your children**	49		51	
**Depression among respondents**				
**Severity of depression**	**Frequency( in percentage)**			
**Minimal depression**	29			
**Mild depression**	39			
**Moderate depression**	23			
**Moderately severe depression**	6			
**Severe depression**	3			

In Table 3, a cross tabulation of depression with the other studied factors is presented to show possible significant relationships. Chi square test was done. The severity of depression was found to be highly significant when cross tabulated with respondent educational status (p value = 0.003), inability to attend social gatherings (p value = 0.001), and drug intake to relieve tension (p value = 0.000). Parents who were more depressed were taking drugs to relieve tension(p value 0.000). As the educational status of the respondent increased, the severity of depression increased.(p value 0.003). No significant relationship was found between the depression of a parent and the birth order of the affected child (p value 0.084). The severity of depression was not different among the parents grouped by age. (p value 0.0167).

**Table 3:** Cross tabulation of depression with other studied factors (percentages given)

	**Severity of Depression**					*P value
	**Minimal**	**Mild**	**Moderate**	**Moderately Severe**	**Severe**	
**Respondent’s Age**						
15 to 20	0	2	0	0	0	0.167
20 to 25	1	6	0	0	0	
25 to 30	13	12	6	0	3	
30 to 35	3	8	5	1	0	
35 to 40	8	8	7	3	0	
Above 40	4	3	5	2	0	
**Respondent’s Gender**						
Male	10	12	3	0	0	0.145
Female	19	27	20	6	3	
**Birth Order of Affected Child**						
1^st^	11	16	7	1	3	0.084
2^nd^	9	9	4	3	0	
3^rd^	1	4	4	0	0	
4^th^	5	8	1	0	0	
5^th^	1	1	6	2	0	
6^th^	2	1	0	0	0	
Above 6^th^	0	0	1	0	0	
**Respondent’s Education**						
Illiterate	15	31	19	3	0	0.003
Undermatric	7	3	2	2	3	
Matric	7	2	0	1	0	
Undergraduate	0	2	0	1	0	
Postgraduate	0	1	0	0	0	
**Take drug to relieve tension**						
Yes	4	5	7	6	1	0.000
No	25	34	16	0	2	
**Unable to attend Social Gatherings**						
Not at all	12	4	4	0	0	0.001
Slightly	9	12	3	4	0	
Moderately	4	9	10	0	0	
Excessively	4	14	6	2	3	

*Chi square test was applied for significance testing.

## Discussion

The existence of a life threatening or long-lasting disease in a child is a condition that causes stress in parents and that can predispose them to psychosocial disorders.

The comparison of the results of our study with a 2004 study conducted in Iran on “Depression in mothers of children with thalassemia” by Sharghi et al. revealed that 71 percent of the parents in our study had depressive illness while the rate was 51 percent in the Sharghi study. The high percentage of depression in our study might be due to the small sample size. The high depression in these people is due to uncertainty about the future of their child, criticism by relatives, and some level of conflict with their spouse. In our study, 23 percent had conflicts with their spouse, while the study in Iran showed this rate to be 14.1 percent. This may be due to a lack of education and gender bias in our country. People have the misconception that this disease arises due to the bad blood of the mother. No significant relationship was found in age or depression in the parents of our study (p value = 0.167), but there was an inverse relationship between depression and age of the mother in the study conducted in Iran (p value = 0.001) [9].

Comparison with a study conducted in India showed that the psychiatric problems are high 71 percent in our participants, as compared to their 51 percent. 16 percent of our patients showed excessive sleep problems, while the Indian study revealed only 1 percent of the parents had such problems [10].

As this study was a questionnaire based study, there is a possibility of subjective bias. , which could be a reason for the high level of depression among our respondents. One of the limitations of this study was the lack of comparison with healthy controls. The inclusion of a control group would give a more convincing assessment of the psychosocial aspects and would indentify confounding factors for various socio- demographic variables.

The high psychosocial morbidity of parents of thalassemic children due to chronicity of disease highlights the need to focus more attention on parents of thalassemic children. The fact that this is a disease of early childhood has a great psychological effect on the parents. There is lack of education among parents and the general public that leads to many social problems. To address these problems a holistic approach must be used. Awareness about this disease should be increased through health education. Counseling facilities for parents should be available at each thalassemic centre in Pakistan. Parent counseling is needed on a regular basis. Prenatal diagnosis should be available at each thalassemia centre, as the majority of parents in our study were willing to terminate their pregnancy in case of a prenatal diagnosis of thalassemia.

## Abbreviations

PHQ-9: Patient Health Questionnaire 9; PKRs: Pakistani Rupees; QOL: Quality of Life.

## Competing interests

The authors declare that they have no competing interests.

## Authors’ contributions

KA designed the study, helped in data collection, did the literature review, and drafted the manuscript. BS conceived of this study, did data collection, performed the statistical analysis, and helped in drafting the manuscript. SK designed the questionnaires and helped with the statistical analysis. All authors read and approved the final manuscript.

## Supplementary Material

Additional file 1 Psychosocial problems of parents of thalassemic children attending. Click here for file
