# Supplementary material for: Psychosocial problems of Pakistani parents of Thalassemic children: a cross sectional study done in Bahawalpur, Pakistan
Source: Biopsychosoc Med. 2012 Aug 1;6:15. doi: 10.1186/1751-0759-6-15 (PMC3489523; doi:10.1186/1751-0759-6-15)
Supplement: Additional file 1 — Psychosocial problems of parents of thalassemic children attending. [file 1751-0759-6-15-S1.doc]

***Annexure- I***

**PSYCHOSOCIAL PROBLEMS OF PARENTS OF THALASSEMIC CHILDREN ATTENDING**

**Thalassemia Centre, Bahawal Victoria Hospital Bahawalpur**

**Consent**: Are you willing to answer some question? Your answer will be kept confidential and used only for research. Yes No Signature: __________________________________________________

**Name:_______________________________ S/O D/O:________________________________________**

**Age:____________ Gender:____________ Residential address:____________________________**

**Educational status:**

**Father:**  illiterate under matric Matric Undergraduate Graduate Post Graduate

**Mother:**  illiterate under matric Matric Undergraduate Graduate Post Graduate

**Income Status (PKRs)**:<5000 5000-10000 10000-20000 >20000

**Total No of Children**: Male ____ Female______ **No of Affected Children:** Male____ Female_______

**Birth order of affected children**:_________________

1. **Do you have disturbed feelings?** Yes no
2. **Are you able to concentrate on your day to day work?**

Not at all slightly moderately excessively

1. **Has this affected your eating habit?**

Not at all slightly moderately excessively

1. **Has this affected your Sleep pattern?**

Not at all slightly moderately excessively

1. **Is it affecting your economic status?**

Not at all slightly moderately excessively

1. **Are you unable to attend your social gatherings?**

Not at all slightly moderately excessively

1. **Has this affected your relation with your spouse?**

Yes no

1. **Are you being downgraded by your relatives?**

Yes no

1. **Do you take any drug or stimulant to relieve tension?**

Yes no

***If yes then, are you taking it on your own or it is prescribed by a psychiatrist?***

Prescribed by a psychiatrist self medicated

1. **Would you have terminated this pregnancy if it had been diagnosed prenatally?**

Yes no

1. **Do you think this disease has affected your desired family size?**

Yes no

1. **Is this your family marriage?**

Yes no

1. **As you have come to know that this disease occurred due to cousin marriage, would you do cousin marriage of your children?**

Yes no

***Depression score as assessed by PHQ-9:_________***
